# Application of exponential smoothing method and SARIMA model in predicting the number of admissions in a third-class hospital in Zhejiang Province

**DOI:** 10.1186/s12889-023-17218-x

**Published:** 2023-11-22

**Authors:** Wanjun Yang, Aonan Su, Liping Ding

**Affiliations:** Medical Records Statistics Office, Zhejiang Provincial People’s Hospital/People’s Hospital of Hangzhou Medical College, 158 Shangtang Road, Gongshu District, Hangzhou City, 310000 Zhejiang Province China

**Keywords:** Exponential smoothing method, SARIMA model, Number of hospital admissions, Model prediction

## Abstract

**Objective:**

To establish the exponential smoothing prediction model and SARIMA model to predict the number of inpatients in a third-class hospital in Zhejiang Province, and evaluate the prediction effect of the two models, and select the best number prediction model.

**Methods:**

The data of hospital admissions from January 2019 to September 2022 were selected to establish the exponential smoothing prediction model and the SARIMA model respectively. Then compare the fitting parameters of different models: R^2^_adjusted, R^2^, Root Mean Square Error (RMSE)、Mean Absolute Percentage Error (MAPE)、Mean Absolute Error(MAE) and standardized BIC to select the best model. Finally, the established model was used to predict the number of hospital admissions from October to December 2022, and the prediction effect of the average relative error judgment model was compared.

**Results:**

The best fitting exponential smoothing prediction model was Winters Addition model, whose R^2^_adjusted was 0.533, R^2^ was 0.817, MAPE was 6.133, MAE was 447.341. The best SARIMA model is SARIMA(2,2,2)(0,1,1)_12_ model, whose R^2^_adjusted is 0.449, R^2^ is 0.199, MAPE is 8.240, MAE is 718.965. The Winters addition model and SARIMA(2,2,2)(0,1,1)_12_ model were used to predict the number of hospital admissions in October-December 2022, respectively. The results showed that the average relative error was 0.038 and 0.015, respectively. The SARIMA(2,2,2)(0,1,1)_12_ model had a good prediction effect.

**Conclusion:**

Both models can better fit the number of admissions, and SARIMA model has better prediction effect.

**Supplementary Information:**

The online version contains supplementary material available at 10.1186/s12889-023-17218-x.

## Introduction

In recent years, the number of hospital admissions has increased year by year, resulting in hospital congestion, but also produced many problems. Eyles et al. [1] found that the increase in the number of hospital admissions made the allocation of health resources more urgent. In addition, adverse hospital events also increase with the increase of overcrowding, and further affect patient satisfaction, quality of care, treatment, waiting time, and length of stay [2–8].

Inaccurate estimation of the number of admitted patients may lead to insufficient or wasted resource arrangements. On the one hand, the actual number of admitted patients is greater than the estimated number of admitted patients, resulting in hospital crowding [2], which may lead to delayed transfer of patients requiring emergency medical services to the emergency department [3, 4], and even death [5].On the other hand, the actual number of hospital admissions is less than the estimated number of hospital admissions, resulting in a waste of health resources. It follows that it is crucial to address the problem of inaccurate estimates of admissions.

In order to predict the number of hospital admissions, the author comprehensively considers the influence of many factors, and establishes a proper model to predict the number of hospital admissions in the future by using the changing law of the number of hospital admissions in the past. It is of great significance to predict the number of hospital admissions, and to grasp the dynamic change law of the number of hospital admissions provides a basis for rational allocation of health resources [9], and also helps to improve the quality of medical services [10].

At present, most researches on hospital prediction focus on nosocomial infection [11], disease diagnosis [12], disease diagnosis and treatment results [13], disease death [14], disease triage [15], pharmacy service fee [16], blood collection quantity [17], outpatient number prediction [18] and etc. However, few studies have been published on predicting the number of new hospital admissions. Therefore, different time series models were used in this study to predict the number of new hospital admissions. The correct prediction of the number of admitted patients can provide references for the rational allocation of health resources in medical institutions, avoid the insufficiency or waste of medical resources, and improve the efficiency and quality of medical services. The following is reported.

## Data and methods

### Data source

The data comes from the medical record management system of a Grade-A hospital in Zhejiang Province, and the data used were monthly admission data, including gender, age, number of beds in the hospital, number of admission, etc. Specifically, the data of the hospital from January 2019 to September 2022 were selected to draw the original sequence map and establish the model, and the data from October to December 2022 were used to verify the fitting effect of the model, and the data were authentic and reliable. The data for this study was accessed on March 10, 2023. This study was conducted in accordance with the guiding principles of the Declaration of Helsinki and was approved by the Ethics Committee of Zhejiang Provincial People's Hospital. All subjects gave written informed consent.

## Research methods

### Model Introduction

#### Model principles and concepts

##### Exponential smoothing prediction model

The basic idea of exponential smoothing method is that the closer to the predicted point in a time series, the greater the effect. The further away from the predicted point, the less effect it has. The weight of different data is weighted according to the distance in time and the weight is decayed exponentially. The method has 3 parameters to control: Alpha, Gamma, and Delta, corresponding to the level at the current point in time, the slope of the trend part, and the seasonal part, respectively. The parameters Alpha, Gamma, and Delta all have values ranging from 0 to 1, and the closer they are to 1, the greater their weight in the prediction [19].

##### SARIMA model

The principle of ARIMA model is to treat the data of the research object as a random sequence according to the passage of time, and then use a mathematical model to describe this sequence [19]. Since SARIMA(p,d,g)(P,D,Q)s, the model is developed on the basis of the ARIMA model. SARIMA model has 7 main parameters: autoregressive order (p), difference order (d), seasonal autoregressive order (q), moving average order (P), seasonal difference order (D), seasonal moving average order (Q) and seasonal cycle length (s). Stationarity is a necessary condition for the establishment of SARIMA model. Stationarity test: Observe the sequence diagram to determine whether the sequence is stationary. Non-stationary time series can be differentiated and seasonally differentiated until it is stable [10].

#### Model selection

##### Exponential smoothing prediction model

The best exponential smoothing prediction model was selected according to the fitting parameters of different models. The larger the R^2^_adjusted and R^2^ were, The smaller the RMSE, MAPE, MAE and standardized BIC values, the better the fitting effect of the model. Whether the residual sequence is white noise is determined according to the Ljung-Box Q test or the residual graph, and the test level α = 0.05. When *P* > 0.05 or the residual sequence values in the residual auto-correlation and partial correlation graphs fall into the confidence interval, it indicates that the residual sequence is classified as white noise sequence. The relevant formula is as follows:

${R}^{2}=1-\frac{{\sum \left(yi-\widehat{{y}_{i}}\right)}^{2}}{{\sum \left(yi-{y}_{i}\right)}^{2}}$, Where, the numerator part represents the sum of the square variance of the true value and the predicted value, and the denominator part represents the sum of the square variance of the true value and the mean value.

${R}^{2}\_adjusted =1-\frac{\left(1-{R}^{2}\right)\left(n-1\right)}{n-p-l}$, R^2^_adjusted offset the effect of sample size on R^2^.

$\mathrm{MAE}=\frac{1}{\mathrm{n}}\sum_{i=1}^{n}\left|\widehat{{y}_{i}}-{y}_{i}\right|$, represents the average of the absolute error between the predicted and true values.

$\mathrm{RMSE}=\sqrt{\frac{1}{n}} \sum_{i=1}^{n}{\left(\widehat{yi}-yi\right)}^{2}$, represents the sample standard deviation of the difference between the predicted and true values (called the residual).

$\mathrm{MAPE}=\frac{100\%}{n}\sqrt{\sum_{i=1}^{n}{\left(\widehat{yi}-yi\right)}^{2}}$, represents the relative magnitude (i.e., percentage) of the deviation between the predicted value and the true value.

*y*_*i*_ represents the real observed value, $\overline{{y }_{i}}$ represents the average value of the real observed value, $\widehat{{y}_{i}}$ represents the predicted value, R^2^ is the coefficient of determination,n is the number of samples, and p is the number of features.

$\text{BIC} \!=\! \text{ln}(\text{n})\text{k}\!-\!2\text{ln}(\text{L})$, Where k is the number of model parameters, n is the number of samples, and L is the likelihood function.

##### SARIMA model

The order and parameters of the model are determined according to the autocorrelation graph (ACF) and partial autocorrelation graph (PACF), and the SARIMA model is established accordingly. The model with the largest R^2^adj and R^2^ and the smallest RMSE, MAPE, MAE and standardized BIC values is selected as the optimal model.

#### Model evaluation

##### Exponential smoothing prediction model

The results of statistical analysis of the model in this study were evaluated by the model, that is, Parameters α, γ and δ all have values ranging from 0 to 1, and the closer their values are to 1, the greater their weight in the prediction.

##### SARIMA model

The significance test of the model is used to test the validity of the model, and the Ljung-Box Q test is used to test the residual error of the model for white noise. *P* > 0.05 indicates that the residual error is classified as a white noise sequence. The t statistic test was carried out on the model parameters. *P*<0.05 indicates that the t statistic passes the significance test, indicating that we think the established model is suitable.

#### Model prediction

Both the exponential smoothing prediction model and the SARIMA model use the average relative error to judge the prediction effect of the model. the average relative error = $\frac{\sum \left|Predicted Value-Actual value\right|}{\sum Actual value}$. The smaller the average relative error, the closer the predicted value is to the actual value, the better the prediction effect of the model.

### Statistical software

In this study, Excel2016 was used to organize data, and SPSS22.0 was used to establish and verify the model. The difference was statistically significant with *P* < 0.05.

## Result

### Original sequence diagram

**Fig. 1:**
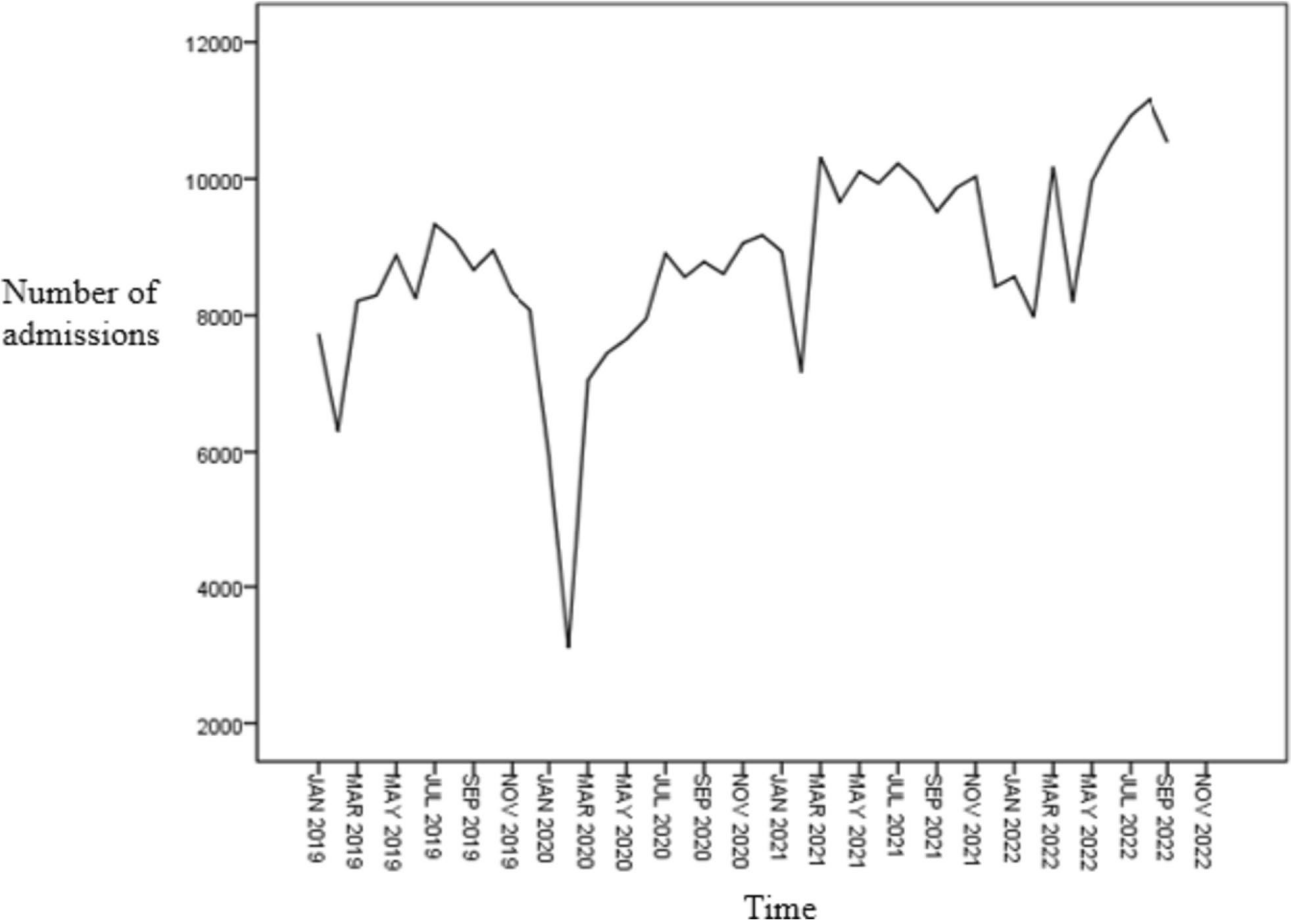
Sequence chart of hospital admissions from January 2019 to September 2022

The number of hospital admissions from January 2019 to September 2022 was sorted out and the sequence chart was drawn. It was found that the number of hospital admissions dropped sharply from January to February each year and rose rapidly in March, showing obvious cyclical changes, as shown in Table 1 and Fig. 1.The number of hospital beds showed an overall upward trend during the study period (Supplement Table 1).

**Table 1:** Number of admissions from January 2019 to September 2022

Time	Number of admissions	Time	Number of admissions
January 2019	7734	January 2021	8935
February 2019	6304	February 2021	7172
March 2019	8212	March 2021	10319
April 2019	8295	April 2021	9662
May 2019	8879	May 2021	10,106
June 2019	8246	June 2021	9931
July 2019	9336	July 2021	10223
August 2019	9093	August 2021	9965
September 2019	8664	September 2021	9515
October 2019	8953	October 2021	9866
November 2019	8332	November 2021	10032
December 2019	8082	December 2021	8419
January 2020	5921	January 2022	8567
February 2020	3103	February 2022	7977
March 2020	7048	March 2022	10178
April 2020	7451	April 2022	8190
May 2020	7654	May 2022	9965
June 2020	7949	June 2022	10504
July 2020	8906	July 2022	10918
August 2020	8559	August 2022	11164
September 2020	8786	September 2022	10533
October 2020	8608		
November 2020	9057		
December 2020	9173

### Model fitting and prediction

#### Exponential smoothing model

##### Model construction and selection

In this study, an expert modeler was used for fitting, and different seasonal exponential smoothing models were used to build models. The fitting parameter results of each model are shown in Table 2. By comparing the fitting parameters of the three models and combining with the evaluation index selection principle, the Winters addition model in the exponential smoothing model was finally determined as the best model. The fitting parameters of this model were the largest in terms of R^2^_adjusted and R^2^ (R^2^_adjusted = 0.533, *R*^2^ = 0.817), and the smallest in terms of MAPE and MAE (MAPE = 6.133, MAPE = 6.133, MAPE = 0.817, MAPE = 6.133, and MAPE = 6.133. MAE = 447.341).

**Table 2:** Model fitting parameters of different seasonal exponential smoothing models and SARIMA models

Model		R^2^_adjusted	R^2^	RMSE	MAPE	MAE	Standardized BIC
Seasonal exponential smoothing models	Simple seasonality model	0.492	0.815	633.212	6.375	476.589	13.071
	Winters addition model	0.533	0.817	636.678	6.133	447.341	13.166
	Winters multiplication model	0.017	0.663	864.776	8.491	634.603	13.779
SARIMA models	SARIMA(2,2,2) (0,1,0)_12_	0.444	0.192	1153.700	8.321	726.972	14.988
	SARIMA(2,2,2) (1,1,0)_12_	0.446	0.195	1177.417	8.333	727.832	15.139
	SARIMA(2,2,2) (0,1,1)_12_	0.449	0.199	1174.694	8.240	718.965	15.134
	SARIMA(2,2,2) (1,1,1)_12_	0.339	0.040	1316.698	10.066	883.491	15.474

**Fig. 2:**
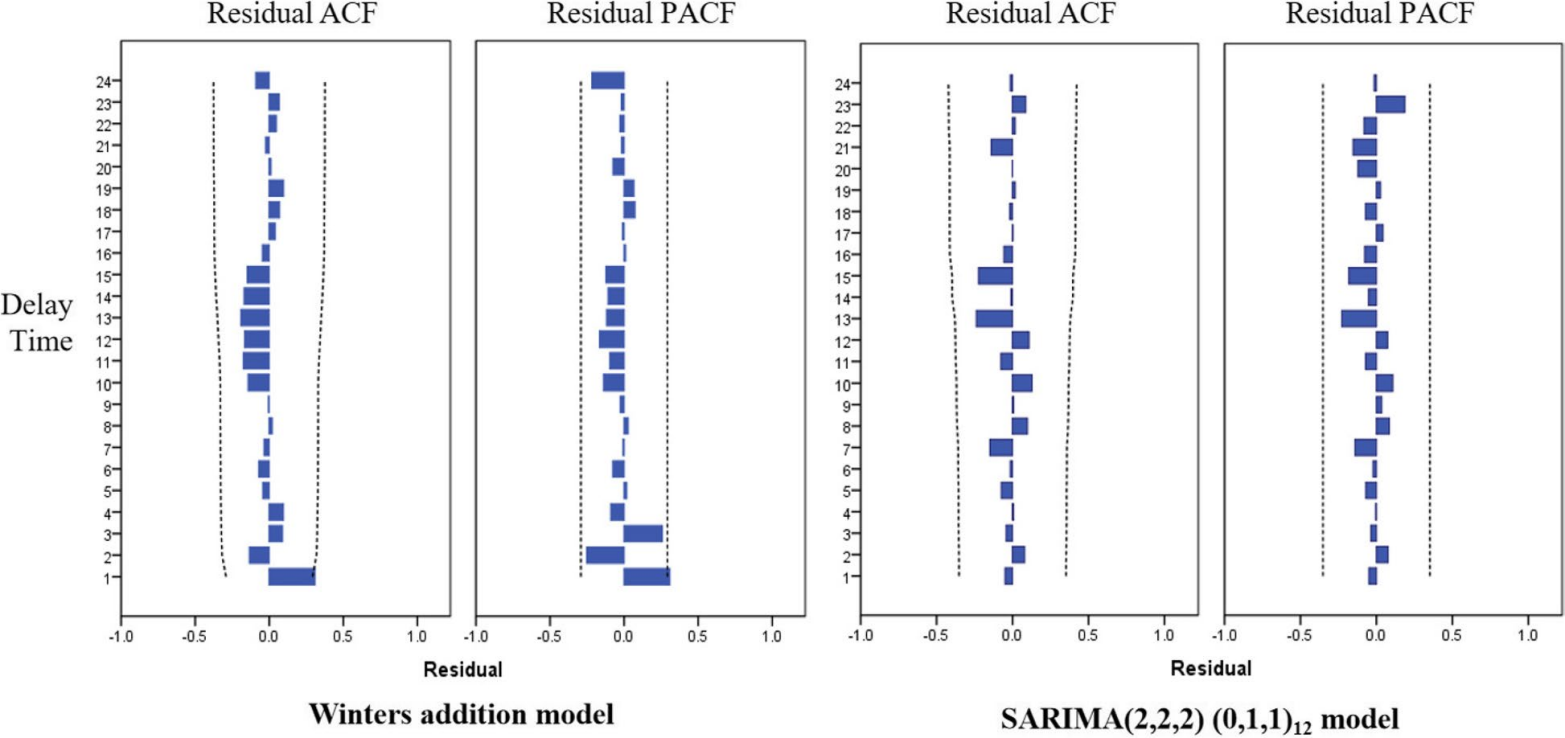
Residual diagram of Winters addition model and SARIMA(2,2,2) (0,1,1)_12_ model

The results of Ljung-Box Q test show that *P* > 0.05, indicating that there is no autocorrelation and partial autocorrelation in the residual sequence after data fitting, and this model can be used for prediction. See Table 3. In addition, it can be seen from the residual autocorrelation and partial correlation graphs that the residual sequence values all fall into the confidence interval, indicating that the model residual sequence does not have autocorrelation and partial autocorrelation, but is a white noise sequence, and the model can extract sequence information adequately. See Fig. 2.

**Table 3:** Ljung-Box Q test results of different exponential smoothing models

Model		Statistical Magnitude	*P* value
Seasonal exponential smoothing models	Simple seasonality model	10.411	0.844
	Winters addition model	13.717	0.547
	Winters multiplication model	18.311	0.247
SARIMA models	ARIMA(2,2,2) (0,1,0)_12_	6.883	0.939
	ARIMA(2,2,2) (1,1,0)_12_	8.824	0.786
	ARIMA(2,2,2) (0,1,1)_12_	10.342	0.666
	ARIMA(2,2,2) (1,1,1)_12_	8.460	0.748

The statistical analysis results of different exponential smoothing models showed that there were statistically significant differences in Alpha(level) among the three models (*P* < 0.05). The simple seasonal model had no statistical significance in Delta(season) (*P* > 0.05), and the Winters addition model and the Winters multiplication model had no statistical significance in Gamma(trend) and Delta(season) (*P* > 0.05). See Table 4.

**Table 4:** Statistical analysis of different exponential smoothing models

Model	Indicator	Estimate	SE	t Value	*P* Value
Simple seasonality model	Alpha	0.700	0.150	4.654	< 0.001
	Delta	1.439	0.351	0.004	1.000
Winters addition model	Alpha	0.700	0.155	4.509	< 0.001
	Gamma	3.852	0.046	0.001	0.999
	Delta	0.001	0.355	0.003	0.998
Winters multiplication model	Alpha	0.335	0.080	4.206	< 0.001
	Gamma	0.001	0.029	0.034	0.973
	Delta	0.269	0.217	1.239	0.222

##### Model prediction

**Fig. 3:**
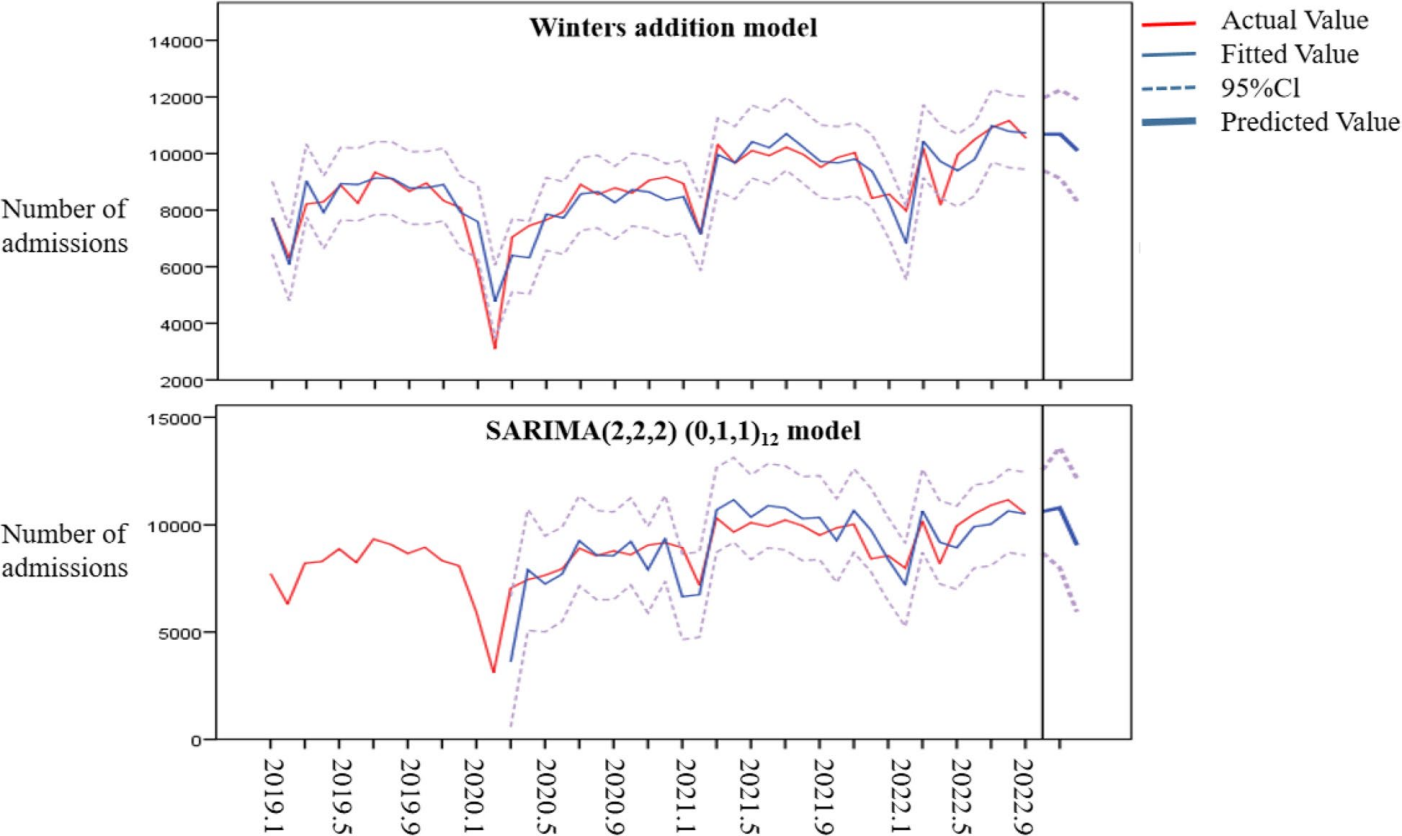
Prediction of hospital admissions in October-December 2022 by the Winters Addition model and SARIMA(2,2,2)(0,1,1)_12_ model

The Winters addition model was used to predict the number of hospital admissions from October to December 2022. The results showed that the average relative error between the predicted value and the actual value was 0.038, and all the predicted number of hospital admissions fell within 95%CI, suggesting that the prediction results were good. The results are shown in Table 5 and Fig. 3.

**Table 5:** Winters' addition model and SARIMA(2,2,2)(0,1,1)_12_ model predicted hospital admissions in October-December 2022

Model	Time	Actual Value	Predicted value	95%CI	Average Relative Error
Winters' addition model	October 2022	10972	10684	9399 ~ 11969	-0.026
	November 2022	10727	10682	9113 ~ 12250	-0.004
	December 2022	8608	10100	9113 ~ 8291	0.173
	Total	30307	31466	-	0.038
SARIMA(2,2,2)(0,1,1)_12_ model	October 2022	10972	10494	8451 ~ 12537	-0.044
	November 2022	10727	10795	7855 ~ 13736	0.006
	December 2022	8608	9484	6254 ~ 12714	0.102
	Total	30307	30773	-	0.015

#### SARIMA Model

##### Sequence stabilization

**Fig. 4:**
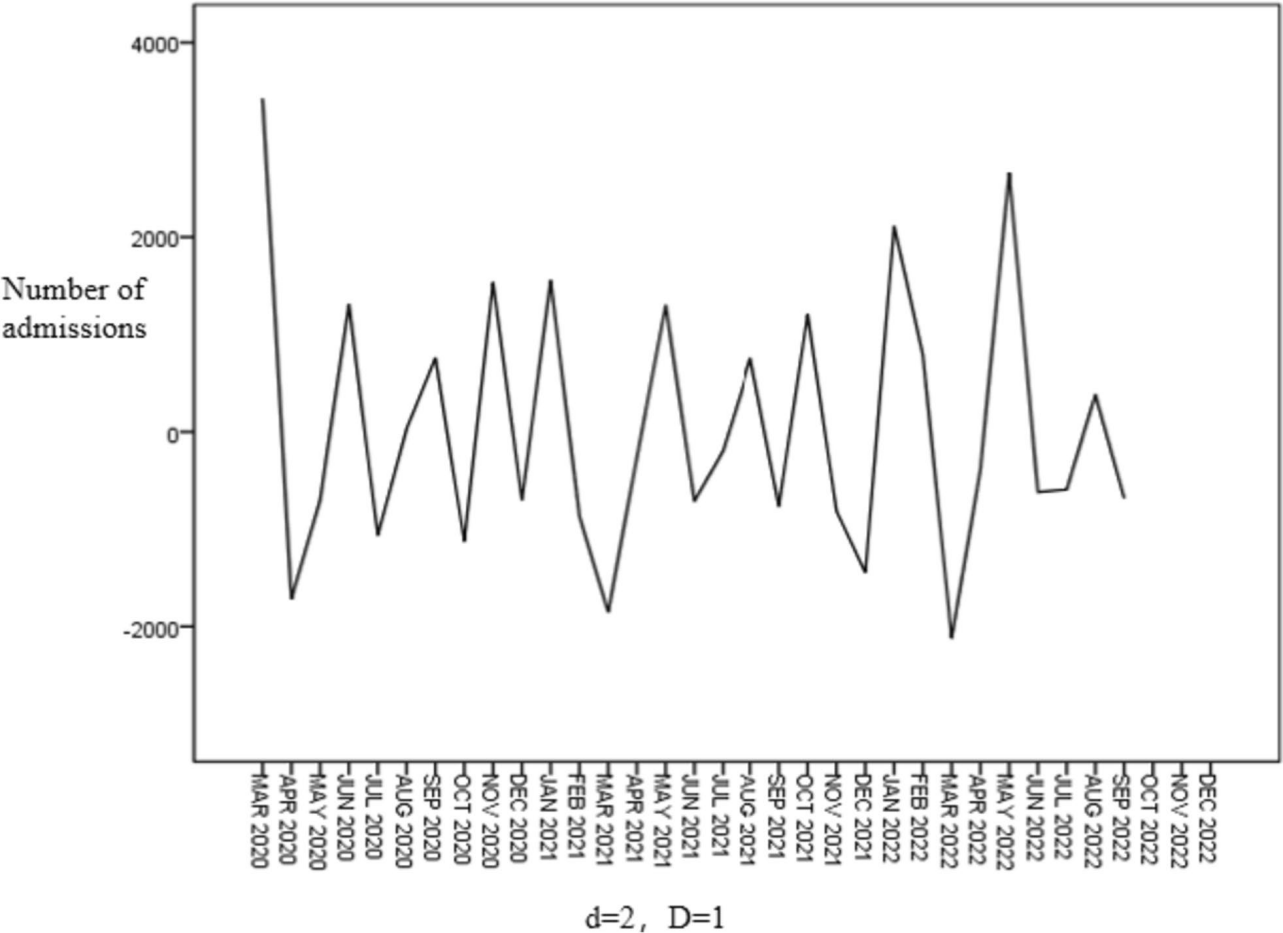
Residual diagram of SARIMA(2,2,2)(0,1,1)_12_ model

It can be seen from the original sequence diagram that the sequence is unstable and periodic. After the trend difference (d = 2) and periodic difference (D = 1), the sequence diagram after the difference observation can be seen to basically stabilize, as shown in Fig. 4.

##### Model recognition

**Fig. 5:**
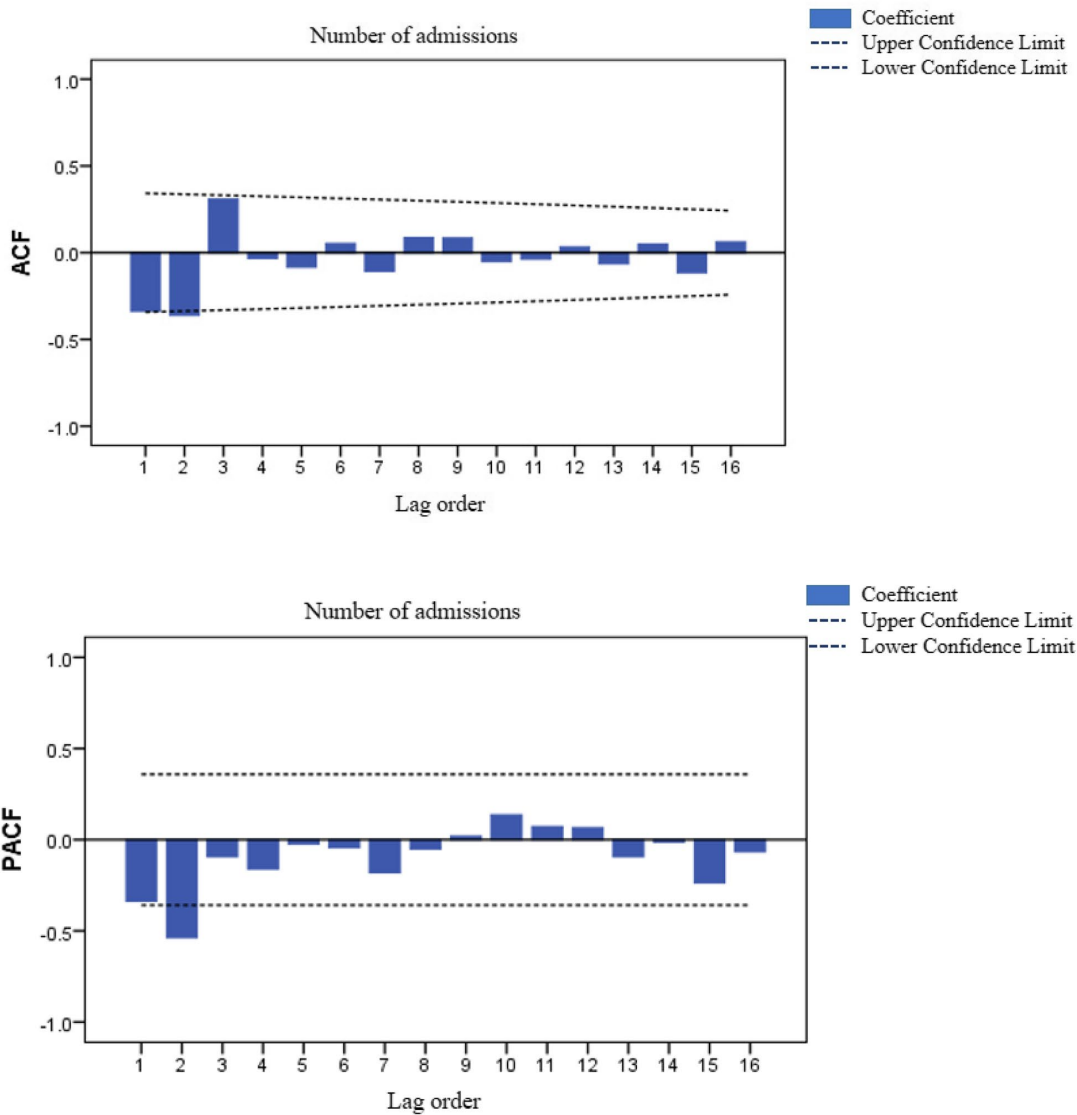
Sequence autocorrelation and partial autocorrelation after difference

According to the results of stabilization, the difference order of the model d = 2, D = 1, the SARIMA (p, 2, q) (P, 1, Q)_12_ model is constructed. According to the autocorrelation function (ACF) diagram and partial autocorrelation function (PACF) diagram of the sequence after difference (Fig. 5). The ACF diagram shows a 2-order truncation, and the PACF diagram shows a 2-order truncation, so q may take 2 and p may take 2. In addition, considering the seasonal autocorrelation characteristics of the series, the ACF plot after difference shows that the delayed 12th-order autocorrelation coefficient is significantly zero, so it can be judged that Q may be 0 or 1. The PACF diagram after difference shows that the partial autocorrelation coefficients of order 12 delay are significantly zero, so P may be 0 or 1.

Then, all possible reasonable models were fitted, and fitting parameters of different models were compared. SARIMA (2,2,2) (0,1,1)_12_ was selected as the best model. The model had the largest R^2^_adjusted and R^2^ (*R*^2^_adjusted = 0.449, *R*^2^ = 0.199), and the smallest MAPE and MAE (MAPE = 8.240, MAE = 718.965). See Table 2.

##### Model diagnosis

Ljung-Box Q test was used to test the residual of the model for white noise, and the results showed no statistical significance (*P* > 0.05), indicating that the null hypothesis of uncorrelated residual should be accepted, and the residual sequence should be classified as pure random sequence and the residual sequence as white noise sequence. SARIMA(2,2,2)(0,1,1)_12_ model is suitable. In addition, it can be seen from the residual autocorrelation and partial correlation that the residual sequence values all fall into the confidence interval, which meets the requirements. See Table 3 and Fig. 2. The t statistic test was conducted for each variable of SARIMA(2,2,2)(0,1,1)_12_ model parameters, and the results showed that the results showed that AR(2),MA(2),MA(2)-periodically and Spring Festival factor t statistics passed the significance test (*P* < 0.05). Therefore, we believe that the established model is appropriate, and the Spring Festival factor is a potential influencing factor for the number of hospital admissions. See Table 6.

**Table 6:** SARIMA(2,2,2)(0,1,1)_12_ Model parameter test

**variable**		**Coefficient**	**SE**	**T**	***P*** ** Value**
Hospital admissions	AR(2)	0.796	0.093	8.546	< 0.001
	d	2			
	MA(2)	0.516	54.629	3.009	< 0.001
	D	1			
	MA(1),periodically	0.632	0.145	4.352	< 0.001
Number of hospital beds		-0.093	1.398	-0.067	0.947
Age		-20.979	220.635	-0.095	0.925
Spring festival factor		-1256.233	402.156	-3.124	0.003

##### Model prediction

The SARIMA(2,2,2)(0,1,1)_12_ model was used to predict the number of hospital admissions from October to December 2022. The results showed that the average relative error was 0.017, and all the predicted values of hospital admissions fell within 95%CI, indicating that the prediction results were good. The results are shown in Table 5 and Fig. 3.

#### Comparison of model prediction effect

The Winters addition model was finally selected for the exponential smoothing prediction model, and the SARIMA model was finally selected for the SARIMA(2,2,2)(0,1,1)_12_ model. The average relative errors of the two models were 0.038 and 0.017 respectively for the number of hospital admissions from October to December 2022, and the prediction effect of SARIMA(2,2,2)(0,1,1)_12_ model is better.

## Discussion

In this study, we established different exponential smoothing prediction models and SARIMA models based on the monthly number of new admitted patients in a hospital to predict the number of new admitted patients in a third-class hospital in Zhejiang Province, and evaluated the prediction effect of the two models. The results show that SARIMA(2,2,2)(0,1,1)_12_ model has the best prediction effect.

In our study, we chose the number of hospital admissions in 2019 and later to build the prediction model, because the outbreak of COVID-19 in 2019 led to a large increase in the number of hospital admissions, and it is not scientific to include the data before the outbreak to predict the number of hospital admissions after the outbreak. Therefore, the prediction models established in this study all start from the year after the outbreak of the epidemic, which is more objective and authentic in terms of data selection.

As shown in Fig. 1, the number of hospital admissions was sorted out and the sequence diagram was drawn. It was found that the number of hospital admissions was significantly lower in January–February each year, which may be due to the Spring Festival holiday. The number of hospital admissions rose rapidly in March, showing obvious cyclical changes. Therefore, we included the Spring Festival factor as a variable in the model to eliminate its influence, and the results showed that the Spring Festival factor was a potential influencing factor for the number of hospital admissions, and the Spring Festival factor was included in the model to better predict the number of hospital admissions (*P* = 0.003). The decrease in the number of hospital admissions during the Spring Festival may be due to the Chinese people's emphasis on the Spring Festival, and people are more inclined to reunite with their families, so that as long as the disease is not particularly urgent, people will not choose to be hospitalized during the Spring Festival, but choose the time before or after the Spring Festival. According to the data of China Influenza Surveillance Information System, the number of outpatient visits to influenza clinics during the Spring Festival was significantly less than that in the week before and after the Spring Festival, mainly due to the holiday in sentinel hospitals, which resulted in a significant reduction in the number of outpatient clinics during holidays [20]. However, other studies have obtained different results. A retrospective cross-sectional study conducted by Wang et al. [21] found that the traditional Spring Festival was associated with hypercholesterolemia, and the serum levels of total cholesterol (TC), triglycerides (TGs), high density lipoprotein cholesterol (HDL-C) and low density lipoprotein cholesterol (LDL-C) of hospitalized patients were higher. This may be due to the irregular life during the Spring Festival, during which people will overeat, stay up late and play. The research results of Zhao et al. [22] showed that the Spring Festival factor was the main reason for the sharp increase in the transmission rate of hand-foot-mouth disease in February. Therefore, in this study, we believe that the Spring Festival factor can be used as a predictor of the number of hospital admissions, which also provides a reference for health resource decision makers to rationally allocate health resources.

In addition, since the number of hospital admissions presents seasonal periodic changes, seasonal factors are considered in the establishment of time series prediction models, and seasonal exponential smoothing prediction models and seasonal SARIMA models are established respectively. The reason for the seasonal variation of the number of hospital admissions may be related to meteorological factors, which mainly include air pressure, temperature, humidity, wind speed, etc. Many reports have shown that meteorological factors can affect the occurrence and progression of diseases, thus increasing the number of hospital admissions.Wang et al. [23] found that high daytime temperature range (DTR) and high relative humidity (RH) exposure increased the risk of hospitalization in patients with rheumatoid arthritis (RA), Ma et al. [24]showed that cold was an important meteorological factor affecting the number of hospitalizations for asthma, and Zhao et al. [22] also found that meteorological factors and population movement had a comprehensive impact on the seasonality of hand-foot-mouth disease transmission in Chinese mainland. The influence of air temperature on diseases may be due to the fact that external temperature will cause changes in human body temperature, thus affecting the body's temperature rhythm, which may affect human immune function and increase the risk of hospitalization for diseases related to human immune function such as rheumatoid arthritis [23]. In addition, humidity may also affect the immune system and increase the level of T cells to increase the risk of hospitalization for diseases related to human immune function, resulting in an increase in the number of admissions [25]. In addition to immunity, the influence of air temperature and temperature on the number of hospital admissions for infectious diseases is also related to the preferred growth environment of pathogenic bacteria or viruses, such as low temperature and low radiation/sunlight, which is conducive to the survival of coronavirus, which means that coronavirus pneumonia may occur in high frequency in winter [26]. Enteroviruses like hot and humid environments, so hand-foot-mouth disease is usually high in summer [27].

As mentioned in related literature [28, 29], RMSE is not always superior to MAE parameters, and a combination of indicators is usually required to accurately evaluate model performance. The model with the largest R^2^adj and R^2^ and the smallest MAPE, MAE and standardized BIC is the optimal model. Among the three exponential smoothing prediction models, the Winters addition model was the best fit. Among the four SARIMA models, and the best fitting SARIMA model was SARIMA(2,2,2)(0,1,1)_12_ model. We used the Winters addition model and the SARIMA(2,2,2)(0,1,1)_12_ model to predict the number of admissions in October-December 2022, respectively. We found that the SARIMA(2,2,2)(0,1,1)_12_ model had a better prediction effect, and the average relative error between the predicted value and the actual value of the model was smaller. The predicted value is closer to the actual value. The prediction effect of different models is different in different study data. Guo Zaijin et al. [30] believed that both SARIMA model and Holt-Winters exponential smoothing method could better fit the number of tuberculosis cases, and the SARIMA model had better prediction effect, which was consistent with our research results. However, the results of the study of Bien Zilong et al. [31] showed that the relative error sum (0.292) of the predicted value of Holt-Winters additive exponential smoothing was smaller than that of ARIMA product seasonal model (0.402), and the Holt-Winters additive exponential smoothing model was more suitable for short-term prediction of tuberculosis epidemic in Shanghai.

In addition, looking at the three months of October-December 2022 separately, we find that the predictions for October and November 2022 are good (mean relative error < 0.1), whether using the Winters addition model or the SARIMA(2,2,2)(0,1,1)_12_ model. The prediction effect in December 2022 is relatively poor (average relative error > 0.1), which may be due to the widespread infection of the novel coronavirus pneumonia in Zhejiang Province and even the whole of China due to the nationwide release of the prevention and control of the novel coronavirus pneumonia in December 2022. At the peak of infection, some experts pointed out that the infection rate of the new coronavirus pneumonia was as high as 90%. Therefore, the Chinese government has proposed to reduce gatherings and avoid going to hospitals as much as possible, and as a result, the number of hospital admissions in December 2022 has decreased compared to normal months. However, both ARIMA product season model and exponential smoothing model are based on historical data, and the modeling premise is the extension of data. If external influencing factors suddenly change or new variables are introduced, the prediction effect of the model will be greatly affected and the prediction efficiency will be reduced [31]. Therefore, these two models are more suitable for short-term prediction of time series [32, 33]. For further prediction of the series, it is necessary to timely update the data, add new actual values to correct the model, and then re-fit the prediction.

In order to explore the change trend of the number of hospital admissions of different genders, we conducted a stratified analysis of different genders, and the results showed that the models with the best prediction effect for males and females were SARIMA(2,2,2)(0,1,0)_12_ model and SARIMA(2,2,2)(0,1,1)_12_ model (Supplement Table 2, Supplement Table 3), respectively. This indicates that gender has a certain influence on the establishment of the prediction model for the number of hospital admissions. Zafeiridi et al. [34] found that among dementia patients, women were less likely to be admitted to hospital. In this study, the number of hospital admissions for men and women was not exactly the same, which suggests that future prediction of hospital admissions should consider modeling men and women separately in order to accurately estimate the number of hospital admissions for different genders.

In this study, the number of hospital beds could not predict the number of inpatients, possibly because there was no direct correlation between the number of hospital beds and the number of inpatients [35]. To a certain extent, even if the number of hospital beds was small, the mobility of patients would be large, which would not cause hospital crowding. Daily variations and uncertainties in the number of patients arriving or leaving a hospital system can profoundly affect hospital overcrowding and the quality of health services [36]. Therefore, hospital health policy makers need to consider a variety of factors other than the number of beds in the hospital when forecasting admissions, and merely increasing bed capacity is not enough to achieve the forecast and policy goals.

According to the development trend of newly admitted patients, we can make the following suggestions for hospital management. First, hospitals should strengthen the dredging of hospital population flow, and overall control of medical resources according to the daily changes in the number of patients arriving or leaving the hospital system, so as to reduce hospital overcrowding, rational use of medical resources and improve patient satisfaction. Second, the comprehensive overhaul of clinical medical devices and medical facilities should avoid the peak months of hospital admissions as much as possible. In our study, the comprehensive overhaul of medical devices and medical facilities should be carried out in January and February every year. Thirdly, it is suggested to add a time series model to the hospital information system to update and predict the changing trend of the number of admissions in real time.

## Conclusion

We believe that the time series model is suitable for predicting the number of hospital admissions. Compared with the Winters addition model, the SARIMA(2,2,2)(0,1,1)_12_ model has a better prediction effect on the number of admissions, which also provides a basis for the hospital management department to formulate relevant policies and rationally allocate medical resources.

### Supplementary Information

**Additional file 1: Supplement Table 1.** Number of hospital beds from January 2019 to December 2022.

**Additional file 2: Supplement Table 2.** Model fitting parameters of different seasonal exponential smoothing models and SARIMA models.

**Additional file 3: Supplement Table 3.** Seasonal exponential smoothing model and SARIMA model predicted different gender of hospital admissions in October-December 2022.

## Data Availability

The datasets analyzed during the current study are available from the corresponding author on reasonable request.

## References

[1] Eyles E, Redaniel MT, Jones T, Prat M, Keen T (2022). Can we accurately forecast non-elective bed occupancy and admissions in the NHS? A time-series MSARIMA analysis of longitudinal data from an NHS Trust. BMJ Open.

[2] Bahadori M, Teymourzadeh E, Ravangard R, Raadabadi M (2017). Factors affecting the overcrowding in outpatient healthcare. J Educ Health Promot.

[3] Man NWY, Forero R, Ngo H (2020). Impact of the Four-Hour Rule policy on emergency medical services delays in Australian EDs: a longitudinal cohort study. Emerg Med J.

[4] Pförringer D, Breu M, Crönlein M, Kolisch R, Kanz KG (2018). Closure simulation for reduction of emergency patient diversion: a discrete agent-based simulation approach to minimizing ambulance diversion. Eur J Med Res.

[5] Ioannides KLH, Dekker AM, Shin ME, Schriger DL (2022). Ambulances required to relieve overcapacity hospitals: a novel Measure of Hospital strain during the COVID-19 pandemic in the United States. Ann Emerg Med.

[6] McKenna P, Heslin SM, Viccellio P, Mallon WK, Hernandez C, Morley EJ (2019). Emergency department and hospital crowding: causes, consequences, and cures. Clin Exp Emerg Med.

[7] Bao Y, Fan G, Zou D, Wang T, Xue D (2017). Patient experience with outpatient encounters at public hospitals in shanghai: examining different aspects of physician services and implications of overcrowding. PLoS One.

[8] Mh Y, Rezaei F, Haghshenas A, Tavakoli N (2017). Overcrowding in emergency departments: a review of strategies to decrease future challenges. J Res Med Sci.

[9] Hongpeng Wu, Qimin Xiao (2022). Analysis of the seasonal index of the number of inpatients in various specialized wards in a hospital. China Health Statistics.

[10] Zhou L, Zhao P, Wu D, Cheng C, Huang H (2018). Time series model for forecasting the number of new admission inpatients. BMC Med Inform Decis Mak.

[11] Gao Y, Wang C, Wang Y (2022). Establishment and validation of a nomogram to predict hospital-acquired infection in elderly patients after cardiac surgery. Clin Interv Aging.

[12] Shi YC, Zheng Z, Wang P (2022). Development and validation of a novel nomogram to predict chronic total occlusion before coronary angiography. Eur Rev Med Pharmacol Sci.

[13] Raita Y, Goto T, Faridi MK, Brown DFM, Camargo CA, Hasegawa K (2019). Emergency department triage prediction of clinical outcomes using machine learning models. Crit Care.

[14] Jiang A, Shi X, Zheng H (2022). Establishment and validation of a nomogram to predict the in-hospital death risk of nosocomial infections in cancer patients. Antimicrob Resist Infect Control.

[15] Goto T, Camargo CA, Faridi MK, Freishtat RJ, Hasegawa K (2019). Machine Learning-Based Prediction of Clinical Outcomes for Children During Emergency Department Triage. JAMA Netw Open.

[16] Ha D, Song I, Lee EK, Shin JY (2018). Projection of future pharmacy service fees using the dispensing claims in hospital and clinic outpatient pharmacies: national health insurance database between 2006 and 2012. BMC Health Serv Res.

[17] Zhang X, Zhao X, Mou X, Tan M (2021). Mixed time series approaches for forecasting the daily number of hospital blood collections. Int J Health Plann Manage.

[18] Shao Y, Xu J, Qiao Y, Shao Y, Fei JM (2020). The effects of temperature on dynamics of psychiatric outpatients. Front Psychiatry.

[19] Zaijin G, Hao G, Luojing Z. SARIMA model and Holt-Winters index smoothing method in predicting the number of tuberculosis cases in Jiangsu Province. Dis Surveill. 2022;37(8):1042–7.

[20] Chen Tao, Yang Jing, Wang Lijie, et al. Effect of national holidays on the proportion of influenza-like cases. Chinese J Epidemiol. 2018;39(08):1100–5.

[21] Wang D, Zou Y, Li H (2021). Data mining: traditional spring festival associated with hypercholesterolemia. BMC Cardiovasc Disord.

[22] Zhao J, Hu X (2019). The complex transmission seasonality of hand, foot, and mouth disease and its driving factors. BMC Infect Dis.

[23] Coiffard B, Diallo AB, Mezouar S, Leone M, Mege JL (2021). A tangled threesome: circadian rhythm, body temperature variations, and the immune system. Biology (Basel).

[24] Ma R, Zhang G, Kong Y, Jia S (2023). Regional heterogeneity in short-term associations of meteorological factors, air pollution, and asthma hospitalizations in Guangxi. China Public Health.

[25] Gao X, Colicino E, Shen J (2019). Impacts of air pollution, temperature, and relative humidity on leukocyte distribution: an epigenetic perspective. Environ Int.

[26] Nichols GL, Gillingham EL, Macintyre HL (2021). Coronavirus seasonality, respiratory infections and weather. BMC Infect Dis.

[27] Coates SJ, Davis MDP, Andersen LK (2019). Temperature and humidity affect the incidence of hand, foot, and mouth disease: a systematic review of the literature - a report from the International Society of Dermatology Climate Change Committee. Int J Dermatol.

[28] Willmott CJ, Matsuura K (2005). Advantages of the mean absolute error (MAE) over the root mean square error (RMSE) in assessing average model performance. Clim Res.

[29] Chai T, Draxler RR (2014). Root mean square error (RMSE) or mean absolute error(MAE)? – arguments against avoiding RMSE in the literature. Geosci Model Dev.

[30] Guo Z, Gong H, Zhou L (2022). Application of SARIMA model and Holt-Winters index smoothing method in predicting the incidence of pulmonary tuberculosis in Jiangsu Province. Dise Surveill.

[31] Bian Z, Zhuo Y, He Z, Zhang F, Cai Q, Jing Wu (2021). Prediction of tuberculosis epidemic in Shanghai by product season model and exponential smoothing model. J Nanjing Med Univ (Nat Sci Edition).

[32] Wang CL, Li YD, Feng W (2017). Epidemiological features and forecast model analysis for the morbidity of influenza in Ningbo, China, 2006–2014. Int J Environ Res Public Health.

[33] Wang P, Peng Y, Yang XB (2018). ARIMA model and Holt-Winters exponential smoothing method to predict influenza-like cases. Wuhan Mod Prev Med.

[34] Zafeiridi E, McMichael A, O'Hara L, Passmore P, McGuinness B. Hospital admissions and emergency department visits for people with dementia. QJM. 2023;hcad232. 10.1093/qjmed/hcad232. [published online ahead of print, 2023 Oct 9]. 10.1093/qjmed/hcad232 PMC10896632 37812203

[35] By the COVID-19 APHP-Universities-INRIA-INSERM Group (2020). Early indicators of intensive care unit bed requirement during the COVID-19 epidemic: a retrospective study in Ile-de-France region, France. PLoS One..

[36] Kakad M, Utley M, Rugkåsa J, Dahl FA (2019). Erlang could have told you so-A case study of health policy without maths. Health Policy.

