# Supplementary material for: Application of exponential smoothing method and SARIMA model in predicting the number of admissions in a third-class hospital in Zhejiang Province
Source: BMC Public Health. 2023 Nov 22;23:2309. doi: 10.1186/s12889-023-17218-x (PMC10664683; doi:10.1186/s12889-023-17218-x)
Supplement: Supplementary file 1 — Additional file 1: Supplement Table 1. Number of hospital beds from January 2019 to December 2022. [file 12889_2023_17218_MOESM1_ESM.docx]

Supplement Table 1 Number of hospital beds from January 2019 to December 2022

| Time | Number of hospital beds | Time | Number of hospital beds |
| --- | --- | --- | --- |
| January 2019 | 2028 | January 2021 | 2189 |
| February 2019 | 2028 | February 2021 | 2189 |
| March 2019 | 2028 | March 2021 | 2189 |
| April 2019 | 2028 | April 2021 | 2225 |
| May 2019 | 2028 | May 2021 | 2231 |
| June 2019 | 2028 | June 2021 | 2231 |
| July 2019 | 2028 | July 2021 | 2237 |
| August 2019 | 2028 | August 2021 | 2237 |
| September 2019 | 2028 | September 2021 | 2225 |
| October 2019 | 1968 | October 2021 | 2225 |
| November 2019 | 1955 | November 2021 | 2217 |
| December 2019 | 1993 | December 2021 | 2237 |
| January 2020 | 2157 | January 2022 | 2237 |
| February 2020 | 2081 | February 2022 | 2237 |
| March 2020 | 2081 | March 2022 | 2237 |
| April 2020 | 2130 | April 2022 | 2237 |
| May 2020 | 2119 | May 2022 | 2230 |
| June 2020 | 2119 | June 2022 | 2230 |
| July 2020 | 2187 | July 2022 | 2232 |
| August 2020 | 2187 | August 2022 | 2231 |
| September 2020 | 2177 | September 2022 | 2231 |
| October 2020 | 2179 | October 2022 | 2231 |
| November 2020 | 2179 | November 2022 | 2231 |
| December 2020 | 2179 | December 2022 | 2233 |
