# Supplementary material for: Application of exponential smoothing method and SARIMA model in predicting the number of admissions in a third-class hospital in Zhejiang Province
Source: BMC Public Health. 2023 Nov 22;23:2309. doi: 10.1186/s12889-023-17218-x (PMC10664683; doi:10.1186/s12889-023-17218-x)
Supplement: Supplementary file 2 — Additional file 2: Supplement Table 2. Model fitting parameters of different seasonal exponential smoothing models and SARIMA models. [file 12889_2023_17218_MOESM2_ESM.docx]

Supplement Table 2 Model fitting parameters of different seasonal exponential smoothing models and SARIMA models

| Gender | Model |  | R^2^_adjusted | R^2^ | RMSE | MAPE | MAE | Standardized BIC |
| --- | --- | --- | --- | --- | --- | --- | --- | --- |
| Male | Seasonal exponential smoothing models | Simple seasonality model | 0.509 | 0.793 | 337.263 | 6.793 | 253.752 | 11.811 |
|  |  | Winters addition model | 0.545 | 0.797 | 338.158 | 6.524 | 235.957 | 11.901 |
|  |  | Winters multiplication model | 0.046 | 0.635 | 453.419 | 8.335 | 315.031 | 12.487 |
|  | SARIMA models | SARIMA(2,2,2) (0,1,0)_12_ | 0.448 | 0.051 | 651.944 | 9.326 | 405.718 | 13.846 |
|  |  | SARIMA(2,2,2) (1,1,0)_12_ | 0.451 | 0.045 | 664.906 | 9.452 | 412.385 | 13.996 |
|  |  | SARIMA(2,2,2) (0,1,1)_12_ | 0.462 | 0.124 | 658.262 | 9.488 | 410.845 | 13.976 |
|  |  | SARIMA(2,2,2) (1,1,1)_12_ | 0.343 | 0.052 | 744.720 | 11.303 | 493.480 | 14.334 |
| Female | Seasonal exponential smoothing models | Simple seasonality model | 0.489 | 0.837 | 306.204 | 6.185 | 234.627 | 11.618 |
|  |  | Winters addition model | 0.521 | 0.843 | 304.924 | 5.920 | 218.108 | 11.694 |
|  |  | Winters multiplication model | 0.043 | 0.697 | 423.270 | 7.956 | 298.958 | 12.350 |
|  | SARIMA models | SARIMA(2,2,2) (0,1,0)_12_ | 0.374 | 0.254 | 575.773 | 8.599 | 371.292 | 13.598 |
|  |  | SARIMA(2,2,2) (1,1,0)_12_ | 0.387 | 0.269 | 582.706 | 8.617 | 371.851 | 13.732 |
|  |  | SARIMA(2,2,2) (0,1,1)_12_ | 0.394 | 0.277 | 579.407 | 8.441 | 363.119 | 13.721 |
|  |  | SARIMA(2,2,2) (1,1,1)_12_ | 0.331 | 0.202 | 623.251 | 8.577 | 374.054 | 13.978 |
