# Supplementary material for: Application of exponential smoothing method and SARIMA model in predicting the number of admissions in a third-class hospital in Zhejiang Province
Source: BMC Public Health. 2023 Nov 22;23:2309. doi: 10.1186/s12889-023-17218-x (PMC10664683; doi:10.1186/s12889-023-17218-x)
Supplement: Supplementary file 3 — Additional file 3: Supplement Table 3. Seasonal exponential smoothing model and SARIMA model predicted different gender of hospital admissions in October-December 2022. [file 12889_2023_17218_MOESM3_ESM.docx]

Supplement Table 3 seasonal exponential smoothing model and SARIMA model predicted different gender of hospital admissions in October-December 2022

| Gender | Model | Time | Actual Value | Predicted value | 95%CI | Average Relative Error |
| --- | --- | --- | --- | --- | --- | --- |
| Male | Winters' addition model | October 2022 | 5428 | 5246 | 4564~5929 | -0.034 |
|  |  | November 2022 | 5227 | 5202 | 4406~5998 | -0.005 |
|  |  | December 2022 | 4313 | 4992 | 4097~5887 | 0.157 |
|  |  | Total | 14968 | 15440 |  | 0.032 |
|  | SARIMA(2,2,2)(0,1,0)_12_ model | October 2022 | 5428 | 5200 | 4040~6359 | -0.042 |
|  |  | November 2022 | 5227 | 5289 | 3679~6900 | 0.012 |
|  |  | December 2022 | 4313 | 4458 | 2681~6236 | 0.034 |
|  |  | Total | 14968 | 14947 |  | 0.001 |
| Female | Winters' addition model | October 2022 | 5547 | 5457 | 4842~6072 | -0.016 |
|  |  | November 2022 | 5502 | 5506 | 4718~6294 | 0.001 |
|  |  | December 2022 | 4290 | 5082 | 4153~6011 | 0.185 |
|  |  | Total | 15339 | 16045 |  | 0.046 |
|  | SARIMA(2,2,2)(0,1,1)_12_ model | October 2022 | 5547 | 5421 | 4460~6382 | -0.023 |
|  |  | November 2022 | 5502 | 5530 | 4033~7027 | 0.005 |
|  |  | December 2022 | 4290 | 4827 | 3153~4827 | 0.125 |
|  |  | Total | 15339 | 15778 |  | 0.029 |
